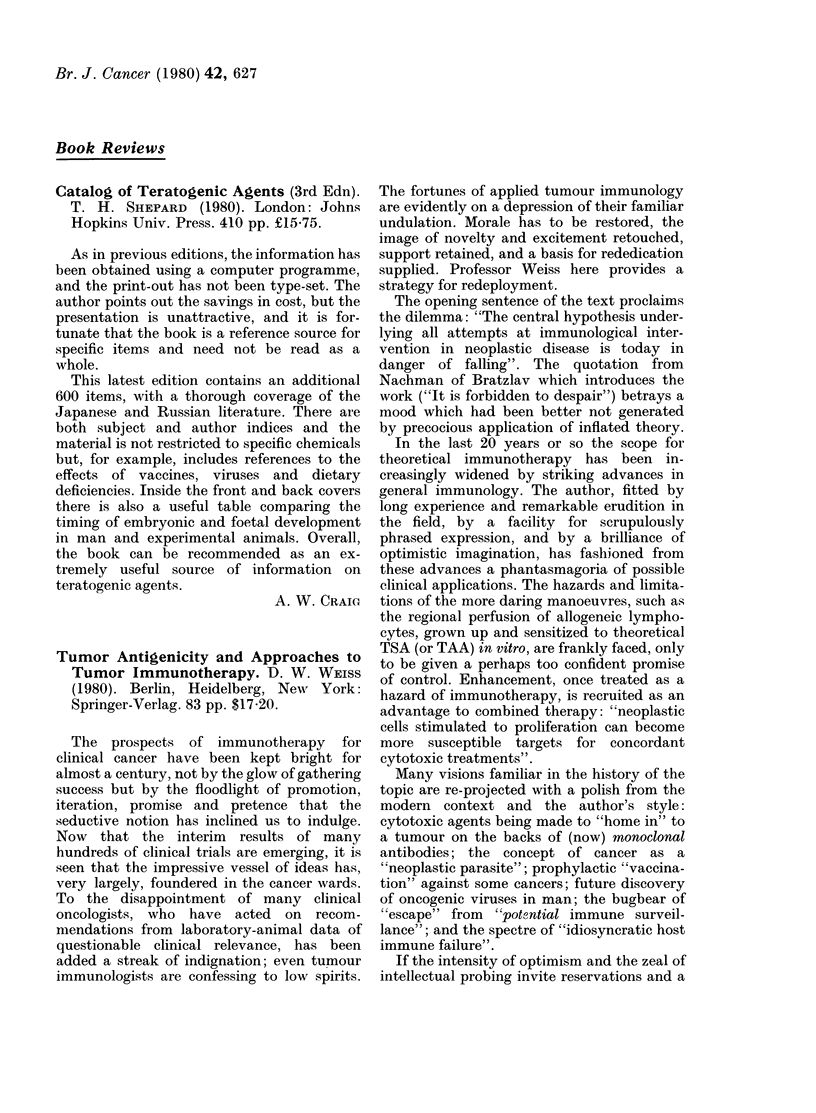# Catalog of Teratogenic Agents

**Published:** 1980-10

**Authors:** A. W. Craig


*Br. J. Cancer* (1980) **42**, 627

### Book Reviews

**Catalog of Teratogenic Agents** (3rd Edn). T. H. Shepard (1980). London: Johns Hopkins Univ. Press. 410 pp. £15·75.

As in previous editions, the information has been obtained using a computer programme, and the print-out has not been type-set. The author points out the savings in cost, but the presentation is unattractive, and it is fortunate that the book is a reference source for specific items and need not be read as a whole.

This latest edition contains an additional 600 items, with a thorough coverage of the Japanese and Russian literature. There are both subject and author indices and the material is not restricted to specific chemicals but, for example, includes references to the effects of vaccines, viruses and dietary deficiencies. Inside the front and back covers there is also a useful table comparing the timing of embryonic and foetal development in man and experimental animals. Overall, the book can be recommended as an extremely useful source of information on teratogenic agents.

A. W. Craig